# Frequency and Significance of Left and Right Diastolic Murmurs

**Published:** 1918-10

**Authors:** 


### ABSTRACTS

**Frequency and Significance of Left and Right Diastolic Murmurs.** Presse Med., Aug. 9, 1917. In young subjects, the murmur of aortic insufficiency is heard more often in the 3d space, to the left, than in the classic position, the 2d right space. As corroborated by radioscopy, the left sided murmur corresponds to a recent lesion, with simple hypertrophy of the left ventricle and a vertical direction of the aorta. In right sided murmurs, the lesion is old, the ventricular hypertrophy is associated with dilatation of the right cavities and the aortic axis inclines from below, to the left, to above, to the right.

